# Linear Representation of Emotions in Whole Persons by Combining Facial and Bodily Expressions in the Extrastriate Body Area

**DOI:** 10.3389/fnhum.2017.00653

**Published:** 2018-01-10

**Authors:** Xiaoli Yang, Junhai Xu, Linjing Cao, Xianglin Li, Peiyuan Wang, Bin Wang, Baolin Liu

**Affiliations:** ^1^School of Computer Science and Technology, Tianjin Key Laboratory of Cognitive Computing and Applications, Tianjin University, Tianjin, China; ^2^Medical Imaging Research Institute, Binzhou Medical University, Yantai, China; ^3^Department of Radiology, Yantai Affiliated Hospital of Binzhou Medical University, Yantai, China; ^4^Research State Key Laboratory of Intelligent Technology and Systems, National Laboratory for Information Science and Technology, Tsinghua University, Beijing, China

**Keywords:** emotion, extrastriate body area, pattern similarity analysis, MVPA, fMRI

## Abstract

Our human brain can rapidly and effortlessly perceive a person’s emotional state by integrating the isolated emotional faces and bodies into a whole. Behavioral studies have suggested that the human brain encodes whole persons in a holistic rather than part-based manner. Neuroimaging studies have also shown that body-selective areas prefer whole persons to the sum of their parts. The body-selective areas played a crucial role in representing the relationships between emotions expressed by different parts. However, it remains unclear in which regions the perception of whole persons is represented by a combination of faces and bodies, and to what extent the combination can be influenced by the whole person’s emotions. In the present study, functional magnetic resonance imaging data were collected when participants performed an emotion distinction task. Multi-voxel pattern analysis was conducted to examine how the whole person-evoked responses were associated with the face- and body-evoked responses in several specific brain areas. We found that in the extrastriate body area (EBA), the whole person patterns were most closely correlated with weighted sums of face and body patterns, using different weights for happy expressions but equal weights for angry and fearful ones. These results were unique for the EBA. Our findings tentatively support the idea that the whole person patterns are represented in a part-based manner in the EBA, and modulated by emotions. These data will further our understanding of the neural mechanism underlying perceiving emotional persons.

## Introduction

The ability to interpret emotions in other people is a crucial social skill in our daily lives. An emotion can be perceived by observing faces, hand gestures, bodies, whole persons, voices, and complex scenes. We know little about emotion perception in the human brain, especially the neural mechanism underlying human body perception. Previous studies have investigated the neural basis of integrating object parts into whole objects (Macevoy and Epstein, 2009), or the combining of two associated objects into an object pair (Baeck et al., 2013). Behavioral studies have shown that the intact bodies can be visually perceived better than the body parts (Soria Bauser and Suchan, 2013). However, the use of static and neutral images in previous studies has limited the interpretation of the data (Liang et al., 2017). Thus, it remains unclear how the combination of faces and bodies is influenced by dynamic emotion information, which may activate just one specific network.

Neuroimaging studies have demonstrated that the stimuli of faces and bodies can activate regions in the ventral (VOTC) and lateral occipitotemporal cortices (LOTC). Faces are represented specifically in two subregions of the VOTC, the occipital face area (OFA) (Pitcher et al., 2007; Liu et al., 2010; Sormaz et al., 2016) and fusiform face area (FFA) (Zhang et al., 2012). Extensive behavioral studies have indicated that human faces are processed in a holistic manner, which means that the featural and configurable information is processed together as an integrated whole (McKone et al., 2001; Maurer et al., 2002). Further functional magnetic resonance imaging (fMRI) studies suggested that the FFA might be engaged in the holistic, non-part-based representation of faces (Zhang et al., 2012), whereas the OFA could process both the features and configurable information of faces (Calder and Young, 2005; Schiltz and Rossion, 2006).

Some similarities between the mechanism of processing bodies and faces (Minnebusch and Daum, 2009) have been confirmed, so the functional contributions of aforementioned face-sensitive areas allow for the understanding of the functional contributions of body-sensitive areas. The bodies or body parts have been found to be represented in the extrastriate body area (EBA) (Downing et al., 2006, 2007; Downing and Peelen, 2016) of the LOTC, and the fusiform body area (FBA) (Schwarzlose et al., 2005; Peelen et al., 2006; Downing and Peelen, 2016) of the VOTC. EBA is found in the posterior end of the inferior temporal sulcus and FBA which partly overlaps the FFA (Peelen and Downing, 2005; Schwarzlose et al., 2005; Peelen et al., 2006; de Gelder et al., 2010) is found in the lateral posterior fusiform gyrus (FG). There is functional similarity between OFA and EBA and between FFA and FBA in some way. By examining whether the perception of bodies was whole- or part-based, one study suggested that the response of the EBA increased linearly with the amount of body-related information (e.g., finger, hand, arm, torso), but in a step-like manner in the FBA, suggesting that the EBA shared a selective role for body parts and the FBA for whole persons or larger body parts (Taylor et al., 2007; Bracci et al., 2015). Furthermore, the FG, which includes the FFA and FBA, could represent the characteristics of the whole person (Kim and McCarthy, 2016). One previous study found that the synthetic patterns which are modeled by a linear combination of face- and body-evoked response patterns could precisely approximate the whole person-evoked response patterns in the right FG, implying a part-based manner of representation (Kaiser et al., 2014). Another recent study suggested that both the EBA and FBA preferred whole bodies to the sums of their scrambled parts (Brandman and Yovel, 2016), which indicated that bodies seemed to be represented in an integrated way, rather than in a part-based way in the EBA and FBA. Therefore, it remains controversial whether the EBA and FG represent the whole person in an integrated or part-based manner.

Some studies have found emotional effects on representations in the EBA (Grezes et al., 2007; Peelen and Downing, 2007) and FG (Fox et al., 2009; Morawetz et al., 2016). FG in the ventral visual stream was suggested to be capable of receiving top-down input signals from regions like the amygdala (AMG) for further detailed processing (Vuilleumier, 2005; Furl et al., 2013; Saarimaki et al., 2016). The superior temporal sulcus (STS) has been identified as playing a selective role in perceiving faces and bodies by fMRI techniques in macaque (Tsao et al., 2003; Pinsk et al., 2005) and human (Tsao et al., 2008; Pinsk et al., 2009) brain studies. Notably, the posterior STS (pSTS) was a crucial node, acting as a hub for processing social stimuli (Lahnakoski et al., 2012). Some studies have demonstrated that the pSTS was involved in the processing of movements, postures, and emotions of faces and bodies (Grezes et al., 2007; Candidi et al., 2011; Zhu et al., 2013; Baseler et al., 2014). In addition, the pSTS, together with the OFA and FFA, was found to comprise a core system of face perception (Fox et al., 2009). The core system for face perception was extended by including the AMG, inferior frontal gyrus (IFG), and insula, which were supposed to be recruited in processing emotional expressions (Ishai et al., 2005). However, emotion perception and experience do not show the 1:1 relationship within each brain region that the model suggests. The AMG, for example, is thought to underlie the decoding of facial expressions, but its activity may be present with other emotions and may at times be absent with fear (Sormaz et al., 2016; Zhang et al., 2016). Therefore, it remains unclear whether these areas could be modulated by emotion when representing the whole person in an integrated or part-based manner.

**FIGURE 1:**
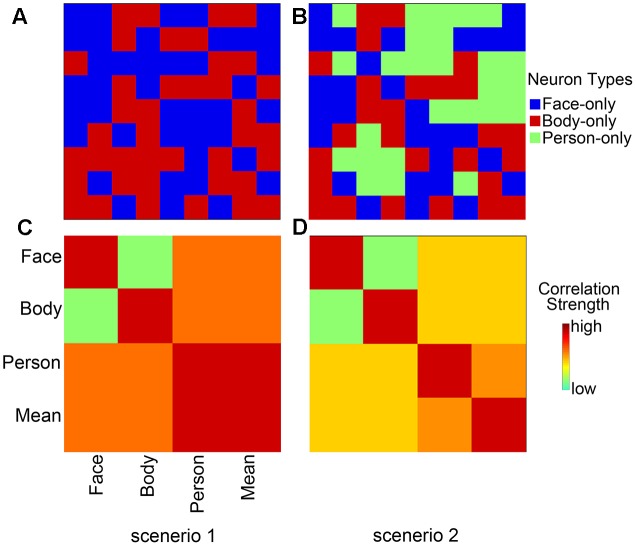
Two hypothetical representation systems. **(A)** Scenario 1, part-based representations are shown as the activities of a population of neurons. Whole person perception activates only face- and body-selective neural populations. **(B)** Scenario 2, integrated representations are shown as the activities of a population of neurons that activate not only face- and body-selective neural populations, but also whole person-only neural populations. **(C)** Scenario 1, part-based representations shown as multi-voxel patterns. Whole person patterns are strongly correlated with the synthetic mean patterns, which were calculated as the average of, face and body patterns. **(D)** Scenario 2, integrated representations shown as multi-voxel patterns. The correlations between whole person patterns and the synthetic mean patterns were much weaker.

In this study, we considered two possible scenarios (**Figure 1**). In the first, whole person perception activates nothing but face- and body-selective neural populations, implying a part-based representation (**Figure 1A**). In the second, not only face- and body-selective neural populations, but also neurons specifically responsive to whole persons are activated; this reflects an integrated representation (Kaiser et al., 2014; **Figure 1B**). However, the coactivated patterns for multiple voxels can now be examined with the development of fMRI data analysis approaches. As compared with the traditional measure of the mean response magnitude, richer information on neural representations can be provided by the voxel-by-voxel activation patterns, and at a finer scale (Haynes and Rees, 2006; Norman et al., 2006; Liang et al., 2017). The two scenarios suggest different predictions for the pattern associations. In the first scenario, there is a strong correlation between the whole person-evoked response patterns and synthetic mean patterns (the average of face- and body-evoked activity patterns); this reflects a part-based representation (**Figure 1C**). In the second scenario, the whole person patterns cannot be modeled by a linear combination of two isolated face and body patterns, reflecting an integrated representation (**Figure 1D**; Kaiser et al., 2014). In the current study, we hypothesized that: (1) there were several specific areas (AMG, IFG, OFA, EBA, STS, FG, and insula) in which the whole person patterns could be modeled by means of face and body patterns, thus reflecting a part-based representation. Furthermore, because these specific areas were suggested to be capable of processing emotional expressions (Haxby et al., 2000; Ishai et al., 2005), we also hypothesized that (2) emotions could modulate the relationship between the whole person and the synthetic mean person. That is to say the correlation value between the whole person and the synthetic mean person is different within each specific emotion. Therefore, we designed a block fMRI experiment in which images of nine conditions (body types: face, body, whole person; emotions: happiness, anger, fear) were presented to participants. Multi-voxel pattern analysis (MVPA) and pattern similarity analysis were conducted to examine how responses to the whole persons were associated with responses to the isolated faces and bodies in all regions of interest (ROIs) for each of the three emotions. Those regions for which encoding is part-based would demonstrate a good approximation between the whole person patterns and the linear combination of face and body patterns. Furthermore, we employed an optimization procedure to determine the optimal weights for combining the face and body patterns into the whole person pattern. In addition, we performed a multi-class classification analysis to quantify how well the activity patterns of face, body, synthetic mean person, and synthetic weighted mean person (the linear combination of face- and body-evoked response patterns, and the total weight of face and body patterns was 1) could be applied for decoding the emotions of whole person patterns.

## Materials and Methods

### Participants

Twenty-four healthy participants were recruited in this study. All participants were right-handed, with normal or corrected-to-normal vision, and all declared having no history of neurological or psychiatric disorders. Four participants were excluded from further analysis due to movement artifacts, so we actually analyzed 20 participants (10 female; mean age 21.8 ± 1.83 years old, range from 19 to 25 years). This study was carried out in accordance with the recommendations of Institutional Review Board (IRB) of Tianjin Key Laboratory of Cognitive Computing and Application, Tianjin University with written informed consent from all subjects. All subjects gave written informed consent in accordance with the Declaration of Helsinki. The protocol was approved by the IRB of Tianjin Key Laboratory of Cognitive Computing and Application, Tianjin University.

### Experimental Stimuli

**FIGURE 2:**
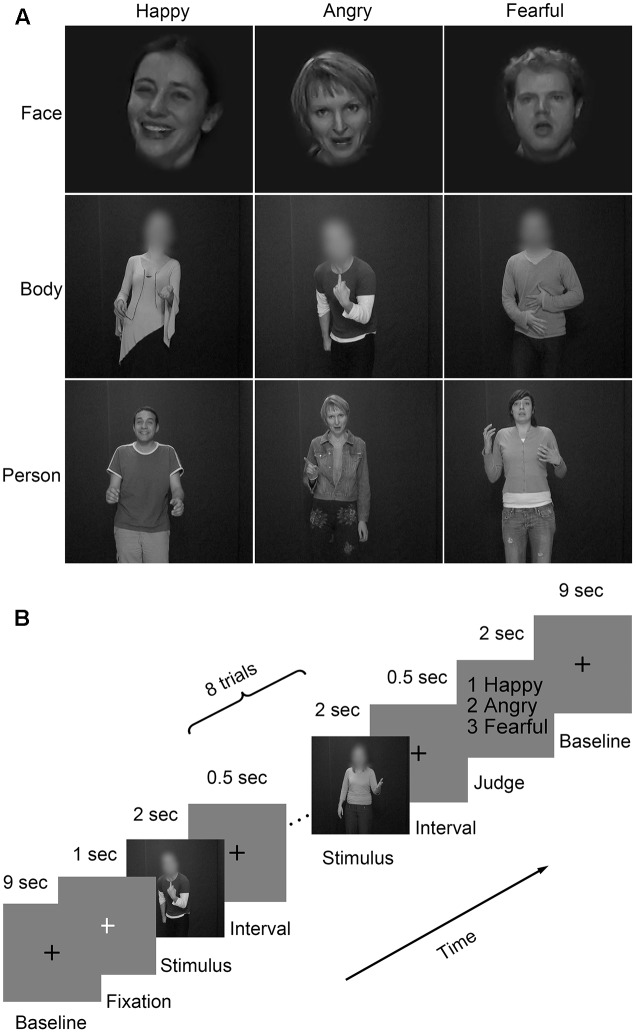
Materials and paradigm of the main experiment. **(A)** Videos of faces, bodies, and whole persons showing three emotions (happiness, anger, and fear) were used in the main experiment. The faces or bodies were masked with Gaussian blur masks; **(B)** Subjects performed four runs of the emotion judgment task. Each of the first three runs consisted of face, body, and whole person conditions, while the last run was merely composed of whole person conditions. A black cross was displayed for 9 s and then a white cross for 1 s to control the attention of the subjects. They were presented between two adjacent blocks. Each block contained eight trials of the same category. Stimuli were presented for 2 s and separated by a 0.5-s interval. At the end of the block, subjects made an emotion judgment task by pressing the corresponding button within a 2-s time limit.

Three emotional materials (happiness, anger, and fear) (Grezes et al., 2007; de Gelder et al., 2012, 2015) were chosen from the GEneva Multimodal Emotion Portrayals (GEMEP) corpus (Banziger et al., 2012). Twenty-four video clips (four male and four female actors × three emotions) were selected and processed in grayscale using MATLAB (Kaiser et al., 2014; Soria Bauser and Suchan, 2015). Videos were edited to a duration of 2000 ms (25 frame/s) by trimming or combining longer- or shorter-length clips, respectively. Adobe Premiere Pro CC 2014 was used to generate the face and body videos by cutting out and masking the irrelevant aspect with Gaussian blur masks (Kret et al., 2011b); also, the face clips were magnified when necessary. The resulting clips were resized to 720 × 576 pixels and presented on the center of the screen. Representative stimuli for the main experiment were presented in **Figure 2A**.

Seventy-two video clips were included in the experiment. An initial validation study was conducted with another group of participants (8 female, mean age: 21.9 years; 10 male, mean age: 22.4 years). Raters were instructed to categorize the emotional materials with six labels (anger, surprise, happiness, sadness, fear, and disgust) and assess the emotional intensities according to a 9-point scale. All expressions were well-recognized (happy face: 97%, angry face: 86%, fearful face: 74%, happy body: 75%, angry body: 93%, fearful body: 82%, happy whole person: 95%, angry whole person: 95%, fearful whole person: 87%). There were no differences in intensity scores between the three emotional expressions [happiness versus anger: *t*(17) = 0.73, *p* = 0.465; happiness versus fear: *t*(17) = 0.26, *p* = 0.796; and anger versus fear: *t*(17) = 1.07, *p* = 0.285].

To examine the quantitative differences in movement in the videos, the movement per clip was estimated by quantifying the variation of light intensity (luminance) between two adjacent frames for each pixel (Grezes et al., 2007; Peelen and Downing, 2007). For each frame, the estimated movements were averaged across the pixels that scored (on a scale reaching a maximum of 255) higher than 10. Subsequently, these scores were averaged for each video. No significant differences were observed between the three emotional expressions [happiness versus anger: *t*(23) = 0.833, *p* = 0.409; happiness versus fear: *t*(23) = 1.639, *p* = 0.108; and anger versus fear: *t*(23) = 2.045, *p* = 0.091]. The low-level visual information of the stimuli, such as the contrast and luminance was also measured. For each frame, the estimated contrast corresponds to the standard deviation of luminance values across the pixels which score reaching a maximum of 255. The root mean square contrast has been shown to be the most reliable indicator of the visibility of broadband filtered images. Subsequently, these scores were averaged for each video. The mean contrast of 72 video clips was 18.89 (*SD* = 7.30). Similarly, the mean luminance of 72 video clips was 24.178 (*SD* = 2.077).

Furthermore, we have compared the luminance and contrast for different emotions and stimulus types. For the contrast, no significant differences were observed between three emotional expressions [happiness versus anger: *t*(23) = 0.304, *p* = 0.763; happiness versus fear: *t*(23) = 0.384, *p* = 0.703; and anger versus fear: *t*(23) = 0.081, *p* = 0.936]. And there were no significant differences for the luminance between three emotional expression [happiness versus anger: *t*(23) = 1.188, *p* = 0.241; happiness versus fear: *t*(23) = 1.188, *p* = 0.241; and anger versus fear: *t*(23) = 0.322, *p* = 0.749]. Statistical analyses for the luminance and contrast between different stimulus types, there was a significant difference between nine stimulus types for the contrast [*F*(23) = 27.382, *p* < 0.001], while no significant difference was observed for the luminance [*F*(23) = 0.613, *p* = 0.764].

### Procedure

There were four runs in the main experiment (**Figure 2B**). For each of the first three runs, three emotions (happiness, anger, and fear) expressed by three body types (face, body, and whole person) were presented. For the last run, only emotions expressed by the whole person were used. There was a 10,000 ms inter-block fixation interval (a black cross presented for 9000 ms and a white cross presented for 1000 ms to control subjects’ attentions). Eighteen blocks of eight trials were pseudo-randomly presented each run. A trial consisted of a 2000 ms video and an inter-stimulus interval (ISI) of 500 ms. At the end of each block, participants were asked to make a choice between three emotions using a button press within a delay of 2000 ms.

The localizer run adopted a block design. Stimuli included four categories of dynamic or static face, body, person, and object. This run contained 16 blocks in total (4 categories × static/dynamic × repeat 2 times), and each type had two blocks, which included eight trials (1.5 s each) and a 10-s interval between blocks. The localizer run lasted for 362 s in total.

### Data Acquisition

Functional images were acquired by a 3.0 T Siemens scanner in Yantai Hospital Affiliated to Binzhou Medical University using an eight-channel head coil. Foam pads and earplugs were used to reduce the head motion and scanner noise. T2^∗^-weighted images were acquired using an echo-planar image (EPI) sequence. In addition, T1-weighted images for an anatomical localization were acquired using a three-dimensional magnetization-prepared rapid-acquisition gradient echo (3D MPRAGE) sequence. The stimuli were displayed by high-resolution stereo 3D glasses within a VisualStim Digital MRI Compatible fMRI system. The imaging parameters of our experiment are provided in **Table 1**.

**Table 1:** Imaging parameters of T2^∗^-weighted and T1-weighted images.

Parameters	TR (ms)	TE (ms)	FOV	Voxel size (mm^3^)	Matrix size	Slices	Thickness (mm)	Slices gap (mm)	FA
T2^∗^-weighted images	2000	30	224 × 224	3.1 × 3.1 × 4.0	64 × 64	33	4	0.6	90°
T1-weighted images	1900	2.52	256 × 256	1 × 1 × 1	256 × 256	176	1	0	9°


*TR, repetition time; TE, echo time; FOV, field of view; FA, flip angle.*

### Data Analysis

#### Behavioral Measures

For each participant, the recognition accuracies and response times for the three emotions were calculated. Accuracies were tested using an analysis of variance (ANOVA) to examine the main effect and interactions between the factor Category and Emotion. Further paired *t*-tests were used to test the differences among the three emotions. SPSS 18 Software was used to perform the statistical analysis.

#### Data Preprocessing

Data preprocessing was performed using the SPM8 software package^1^. The first five volumes of each run were discarded to allow for equilibration effects. The remaining 283 volumes of each run were slice-time corrected, spatially realigned to the first volume, subsampled at an isotropic voxel size of 3 mm, and normalized in the standard Montreal Neurological Institute (MNI) space. Especially for the functional images in the localization run, a 4-mm full-width at half-maximum (FWHM) isotropic Gaussian kernel was used for smoothing. The data in the first four runs were used without smoothing, as this was more suitable for the pattern similarity, weight, and pattern classification analyses. Then a general linear model (GLM) was constructed for each subject, and the subsequent analysis was conducted on each of the first three runs, generating nine activity patterns in total (happy face, happy body, happy whole person, angry face, angry body, angry whole person, fearful face, fearful body, and fearful whole person). Several sources of spurious variances along with their temporal derivatives were removed through the linear regression: six head motion parameters and averaged signals from white matter and cerebrospinal fluid (Power et al., 2015).

#### Localization of Face- and Body-Selective Regions

The face-, body-, and both-selective regions were defined through a separate localizer run, in which participants performed a one-back task on face, body, whole-person, and object stimuli. The localizer scan consisted of 16 randomized blocks (four categories: face, body, whole person, and object; two statuses: static and dynamic, twice repeated for each condition) of eight trials. Each block was followed by a 10,000 ms fixation interval. Face, body, and whole person videos were the same as those in the main experiment. Object clips were selected from the materials used in a previous study (Fox et al., 2009). The middle static frames of video clips were used as the image stimulus. All stimuli were in grayscale and presented for1400 ms with an ISI of 100 ms on a gray background. Participants were required to indicate whether the present stimulus was the same as the previous one.

**FIGURE 3:**
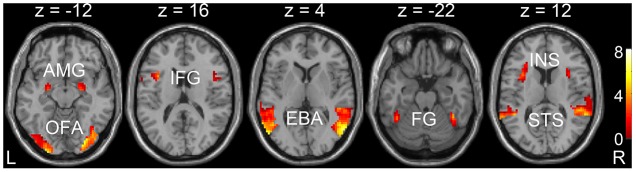
Localizations of regions of interest (ROIs) at the group level. Localization was considered set for an ROI when *p* < 0.05, and with cluster corrections with a minimum extent of 10 voxels. AMG, amygdala; IFG, inferior frontal gyrus; OFA, occipital face area; EBA, extrastriate body area; STS, superior temporal sulcus; FG, fusiform gyrus; INS, insula.

Through the GLM analysis, we identified the face-selective (AMG, IFG, and OFA), body-selective (EBA), and both-selective (STS, FG, and insula) areas by contrasting faces versus objects, bodies versus objects, and the average response to faces and bodies versus objects. The faces, bodies, and objects referred to the average responses to dynamic and static categories. The ROIs were generated with a liberal threshold (*p* < 0.05, with a minimum cluster extent of 10 voxels). The locations of the ROIs were shown in **Figure 3** and **Table 2**.

**Table 2:** The peak coordinates, number of voxels, and peak intensities of functional ROIs.

Hemisphere	Functional ROI	MNI coordinates			Voxel	Peak
				
		*x*	*y*	*z*	number	intensity
R	Amygdala	24	-9	-12	32	2.81
L		-24	-9	-15	35	4.10
R	Inferior frontal gyrus	39	6	33	227	3.56
L		-36	12	15	70	3.76
R	Occipital face area	24	-93	-3	190	5.88
L		-24	-99	-12	149	4.89
R	Extrastriate body area	51	-72	0	879	7.33
L		-54	-72	3	660	5.86
R	Superior temporal sulcus	54	-36	9	429	5.74
L		-45	-45	12	161	4.37
R	Fusiform gyrus	42	-48	-24	56	5.42
L		-42	-51	-21	40	2.92
R	Insula	36	21	3	176	2.45
L		-33	12	15	235	3.33


*Coordinates refer to the MNI coordinate system. *p* < 0.05, uncorrected, with a minimum cluster extent of 10 voxels. L, left. R, right.*

#### Multi-Voxel Pattern Analysis (MVPA)

**FIGURE 4:**
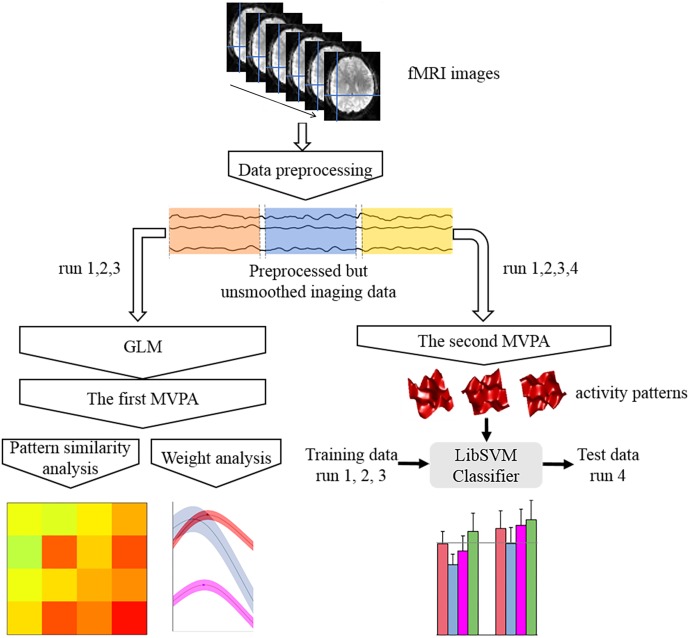
Flow chart of the main analytical steps. There were two kinds of multi-voxel pattern analysis (MVPA) procedures. The results of the first MVPA were applied to pattern similarity analysis and weight analysis, while the results of the second one were used to perform classification analysis.

The response pattern in each condition was calculated using MVPA and subsequently used to conduct the pattern similarity, weight, and pattern classification analyses. Specifically, two kinds of procedures for MVPA were included in this study (**Figure 4**). The first procedure utilized the activation patterns of each condition that were extracted from the beta values of the category regressors. These patterns were then used to perform the pattern similarity analysis and weight analysis (Kaiser et al., 2014). Pattern similarity analysis calculates the correlation coefficients between the face-, body-, whole person-evoked activity patterns, and the synthetic mean patterns (the average of face- and body-evoked activity patterns) in each ROI for the three emotions. The weight analysis can identify the weights of the face and body patterns for the case when the actual whole person patterns are maximally correlated with the synthetic mean patterns; thus, we initially evaluated the relative importance of face part and body part when people recognized a whole person. In the second MVPA step, the activation patterns of each ROI were drawn out from the normalized time series and 283 volumes were used per run. Subsequently, the activation patterns of each condition for every ROI were extracted from the time series and 20 volumes were used per condition per; these activation patterns were then used to perform the pattern classification analysis (Harry et al., 2013). The purpose of pattern classification analysis is to determine which category among the face, body, synthetic mean, and synthetic weighted mean patterns could best decode the emotions expressed by whole persons. It is important to note that only the functional data in the first three runs were used to conduct the pattern similarity analysis and weight analysis, because face, body, and whole person patterns have the same sampling points. However, we used all four runs to perform the category classification analysis to ensure that the training data (the first three runs) and test data (the fourth run) would be independent.

In the pattern similarity analysis, the average of face and body patterns across all voxels in a given ROI was calculated as a synthetic mean pattern, which was similar to the approach used in previous studies (Baeck et al., 2013; Kaiser et al., 2014). To estimate the pattern similarity of different categories, we computed the Pearson’s linear correlations among the face, body, whole person, and synthetic mean patterns for every two out of three runs (three possible pairs altogether), and then a Fisher’s *Z* transformation was conducted. After that, a representational similarity matrix (RSM) was constructed for each individual subject. The RSMs were then averaged at the group level and 2 × 3 ANOVA analyses were performed to examine the main effect and significant interactions between the factors Category and Emotion.

To detect the optimal weights for face and body patterns within each emotion when modeling the whole person pattern, we designed a simple optimization procedure by our own to obtain the maximum value of the correlation between a linear combination of the face and body activation patterns with the whole person pattern, as was done in a previous study (Kaiser et al., 2014). For each subject, the data (the results of the first MVPA procedure) were first averaged across three runs. The optimization procedure was then conducted individually, and a Fisher’s *Z* transformation analysis was performed to transform the Pearson’s correlation values to *z*-values. The total weight of face and body patterns was 1, given that the correlation magnitude was assumed to be related only to the face and body patterns. Hence, our approach represents the relative, rather than absolute, contributions of face and body patterns. We set the face coefficient to α, and the body coefficient to β, such that it was constrained to be identical to (1-α). Thus the synthetic weighted mean pattern was approximately equal to α ^∗^ face pattern + β ^∗^ body pattern. The correlation coefficients varied with the increase of α from 0 to 1 in 0.01 increments. The optimal weights of face and body patterns were obtained when the correlation between the synthetic mean patterns and whole person patterns reached its maximum value. Finally, statistical analyses were conducted on the correlations from the various alpha/beta values for each subject to examine the statistical significance.

If the whole person patterns could be represented by the face and body patterns, we inferred that the whole person patterns could be decoded using the combination of face and body patterns. So the pattern classification analysis using the multi-voxel patterns was carried out to assess the relationship between the whole person pattern and the single part (“face” and “body” pattern) and synthetic patterns (“mean” and “weighted mean” pattern). In MVPA^2^, the functional imaging data were changed into activity patterns that were subsequently transformed to *z*-scores. Then significant feature extractions were conducted using ANOVA (*p* = 0.05) over all of the first three runs and all conditions, which were essential for reducing irrelevant features and achieving good performances (Pereira et al., 2009). By applying a linear support vector machine (LibSVM)^3^ to perform the pattern classification analysis over emotions, we designed a “whole person” SVM predictor (from the fourth run) and four training models (from the first three runs). The models were trained by four patterns: a “face” and a “body” pattern, each evoked by face or body separately; a “mean” pattern that was represented by a combination of face and body patterns at the same weights, and a “weighted mean” at the individual optimal weights that were estimated in the above optimization procedure. It was worth noting that the pattern classifiers were trained or tested separately for each ROI. The classification results were tested against chance (33.33%) at the group level and corrected for multiple comparisons by analyzing the false-discovery rate (FDR) across 28 comparisons (seven ROIs and four classification accuracies for each).

## Results

### Behavioral Performance

The mean recognition accuracy of face, body, and whole person expressions was 98.0% (*SD* = 5.3). The 3 × 3 ANOVA for accuracies with the factors Category (face, body, and whole person) and Emotion (happiness, anger, and fear) revealed no significant main effect for Category [*F*(2,38) = 1.03, *p* = 0.367] or Emotion [*F*(2,38) = 2.98, *p* = 0.063], nor a significant interaction [*F*(4,76) = 0.69, *p* = 0.599]. The statistical analysis by 3 × 3 ANOVA for the response time with the factors Category and Emotion showed a main effect for Emotion [*F*(2,38) = 20.53, *p* < 0.001] but not for Category [*F*(2,38) = 1.91, *p* = 0.162]; nor were any significant interactions observed [*F*(4,76) = 1.91, *p* = 0.118]. Additionally, paired comparisons among the three emotions irrespective of the factor Category showed that subjects’ response times to happy expressions were shorter than those to anger [*t*(19) = 3.98, *p* = 0.001] or fear [*t*(19) = 6.15, *p* < 0.001]. In addition, they reacted significantly faster to the angry expressions than to the fearful ones [*t*(19) = 2.75, *p* = 0.013]. **Table 3** showed the descriptive statistics of behavioral data at the group level. The subjects’ recognition accuracies and response times for the nine conditions in the emotion distinction task were shown, although only the means and standard deviations of correct responses were provided.

**Table 3:** Mean emotion identification accuracies and corresponding response times.

Emotion	Category	Recognition rate (%)		Response time (ms)	
			
		Mean	*SD*	Mean	*SD*
Happy	Face	100	0	713.74	163.39
	Body	97.50	6.11	669.36	160.87
	Person	100	0	675.25	155.35
Angry	Face	97.50	6.11	808.22	235.30
	Body	97.50	6.11	762.83	227.69
	Person	98.75	3.05	767.05	224.22
Fearful	Face	96.67	6.84	809.54	234.73
	Body	96.67	6.84	825.16	220.20
	Person	97.08	5.59	836.10	210.54


### Pattern Similarity Analysis

**FIGURE 5:**
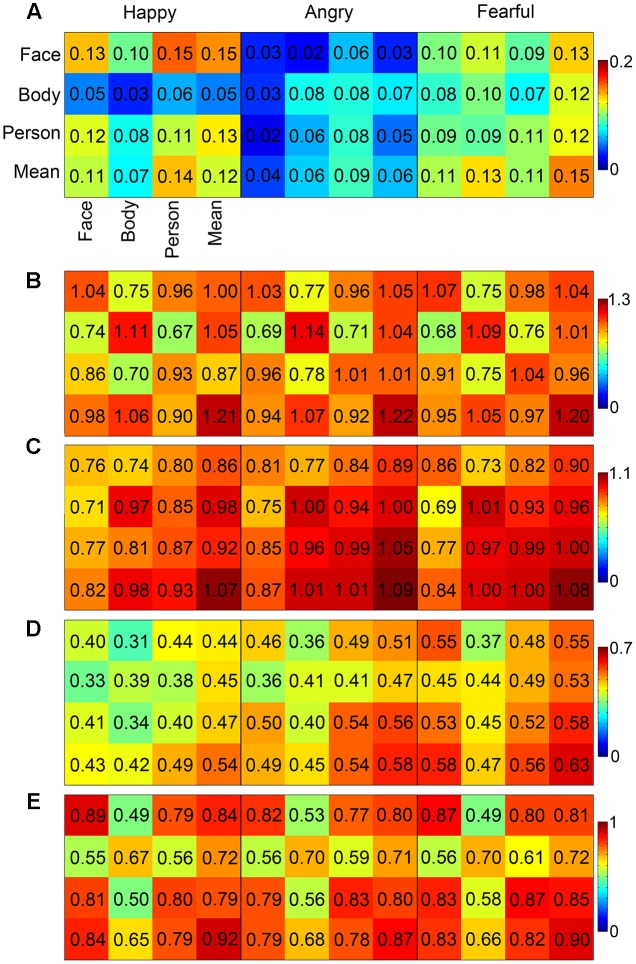
MVPA results. The correlation matrices in the AMG, OFA, EBA, STS, and FG **(A–E)** for the three emotions that were calculated between each pair of the face patterns, body patterns, whole person patterns, and synthetic mean patterns (a simple average of face and body patterns). In the EBA, the whole person patterns can be precisely modeled by the synthetic mean patterns for each emotion.

In order to examine the correlations between the face-evoked patterns, body-evoked patterns, whole person-evoked patterns, and synthetic mean patterns (an unweighted average of face- and body-evoked patterns), a pattern similarity analysis was conducted by calculating the RSM of each ROI. The whole person patterns and the synthetic mean patterns were highly correlated in the OFA, EBA, and FG (*r* > 0.79), and weakly correlated in the STS (happy: 0.48, angry: 0.55, fearful: 0.57). However, the whole person patterns were poorly correlated with the synthetic mean patterns (*r* < 0.32) in the ROIs including the AMG, IFG, and insula. **Figure 5** showed the results of the pattern similarity analyses for face-selective (AMG and OFA; **Figures 5A,B**), body-selective (EBA; **Figure 5C**), and both-selective (STS and FG; **Figures 5D,E**) areas for all three emotion conditions. We also tried to standardize the color scale, but the difference between the patterns of the brain regions became insignificant, as shown in **Supplementary Figure S1**.

Furthermore, we explored whether the whole person patterns were better approximated by the synthetic patterns than by the face or body patterns in the OFA, EBA, FG, and STS. Two 2 × 3 ANOVAs were conducted on the calculated *z*-values. Analysis with the factors Category (person-face and person-synthetic) and Emotion (happiness, anger, and fear) revealed a main effect in the EBA of Category [*F*(1,19) = 20.88, *p* < 0.001], in which the synthetic patterns approximated the person patterns significantly better than did face patterns; however, no significant main effect of Emotion [*F*(2,38) = 1.96, *p* = 0.146] nor significant interaction [*F*(2,38) = 0.18, *p* = 0.834] were identified. In the OFA, FG, and STS, no significant main effects or interactions were found. Analysis with the factors Category (person–body and person–synthetic) and Emotion (happiness, anger, and fear) identified significant main effects of Category in all four brain areas, indicating that the synthetic patterns better approximated the person patterns than did body patterns in all four areas. A main effect for Emotion was also observed in the EBA and STS [EBA: *F*(2,38) = 3.93, *p* = 0.022, STS: *F*(2,38) = 3.56, *p* = 0.032], but not the OFA or FG. No significant interactions were identified for any brain area. Taken together, these results show that only the EBA had greater person–synthetic correlations than both person–face and person–body correlations. In addition, this relationship in the EBA had been modulated by emotion. **Table 4** showed the differences between the person–synthetic correlation and the person–face correlations or the person–body correlations.

**Table 4:** Differences between the person–synthetic correlation and the person–face correlations or the person–body correlations.

Brain areas	The person–synthetic correlation versus the following correlations											
	
	Person–face						Person–body					
		
	Main effect				Interaction		Main effect				Interaction	
				
	Category		Emotion				Category		Emotion			
							
	*F*	*p*	*F*	*p*	*F*	*p*	*F*	*P*	*F*	*p*	*F*	*p*
OFA	0.02	0.880	1.13	0.328	0.19	0.826	30.24	0	1.78	0.174	0.01	0.992
EBA	20.88	0	1.96	0.146	0.18	0.834	4.71	0.032	3.93	0.022	0.12	0.887
STS	2.69	0.104	0.02	0.984	0.02	0.984	15.47	0	3.56	0.032	0.11	0.894
FG	0.03	0.871	0.31	0.733	0.05	0.952	34.37	0	0.65	0.522	0.05	0.949


*OFA, occipital face area; EBA, extrastriate body area; STS, superior temporal sulcus; FG, fusiform gyrus.*

### Weight Analysis

**FIGURE 6:**
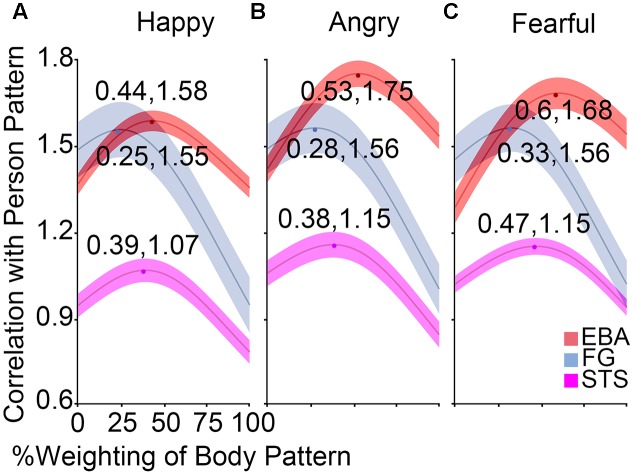
Results of weight analysis. **(A–C)** The correlation curves at the group levels in the EBA, FG, and STS for happy, angry, and fearful emotions, which were computed by the correlations between the whole person pattern and the combination of face and body patterns according to variable weights. The middle line of every band represents the mean optimal estimate, while the half-width of every band represents SEM. The optimal weight of body patterns was lower than that of face patterns for happy expressions in the body-selective EBA, while it was almost the same as that of face patterns for angry and fearful expressions.

To investigate the relative contribution of the face patterns and body patterns in decoding the whole person patterns, an optimization procedure was applied to compute the optimal correlation coefficients. **Figure 6** showed the correlation curves and optimal values in the body-sensitive (EBA) and both-sensitive (FG and STS) areas. The maxima were above 1.55 in OFA, EBA, and FG for any emotion, ranging from 1.06 to 1.15 in the STS and below 0.80 in the other regions for any emotion. At the group level, we examined whether the optimal weights of the body patterns were different for the three emotions in the OFA, EBA, STS, and FG. A 3 × 4 ANOVA for body weighting with the factors Emotion and ROI revealed a significant main effect for Emotion [*F*(2,38) = 10.02, *p* < 0.001] and for ROI [*F*(3,57) = 46.99, *p* < 0.001], although no significant interaction between them was observed [*F*(6,114) = 0.33, *p* = 0.923]. Further paired comparisons showed greater weights of body patterns for fearful expressions than angry [*t*(79) = 13.60, *p* < 0.001] or happy [*t*(79) = 13.80, *p* < 0.001] expressions, while there was no significant difference between angry and happy emotions [*t*(79) = 0.25, *p* = 0.802]. We also discovered that body pattern weights in the EBA were significantly greater than those in the STS [*t*(59) = 4.43, *p* < 0.001], whereas those in the STS were notably higher than those in the FG [*t*(59) = 4.71, *p* < 0.001]. Those in the OFA were the lowest [significantly lower than those in the FG: *t*(59) = 3.63, *p* = 0.001].

Additionally, one-sample *t*-tests in the EBA found that the optimal weights for happy expressions were significantly lower than 0.5 [*t*(19) = 2.99, *p* = 0.008], indicating that more face than body information was needed when combining them to form the whole person pattern. No significant difference from 0.5 was found for the angry stimulus [*t*(19) = 0.42, *p* = 0.676], and only a weak trend toward significance for the fearful stimulus [*t*(19) = 1.80, *p* = 0.088], implying that the whole person patterns could be modeled by a linear combination of half of the face and body patterns in the EBA.

### Pattern Classification

**FIGURE 7:**
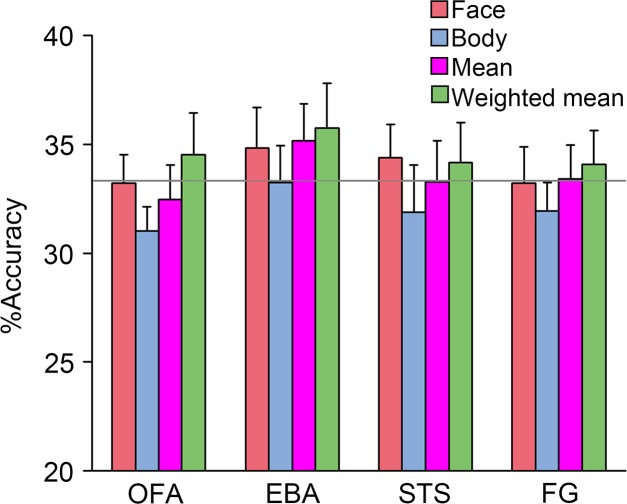
Pattern classification performances. The accuracies of support vector machine (SVM) that were trained by “face,” “body,” “synthetic mean,” and “synthetic weighted mean” and tested by the whole person predictor were not significantly greater than the chance level in all ROIs. Error bars indicate SEM.

If the whole person patterns could be represented by combining the face and body patterns, we inferred that the whole person patterns could also be decoded using the combination. Therefore, four kinds of classification analyzes based on the activated patterns were performed (between three emotions), whose models were trained by the face patterns, body patterns, synthetic mean patterns, and synthetic weighted mean patterns, respectively, and whose predictors were all activity patterns (which were subsequently transformed to *z*-scores) of the whole persons. After FDR corrections for multiple comparisons, none of the face and body patterns were successfully classified in all seven ROIs, which demonstrated that neither part alone could represent the emotional information conveyed by the whole person. Additionally, none of the average patterns or synthetic patterns were successfully classified in any area, while it was worth noting that the two classification accuracies (classifier were trained by the synthetic mean patterns and synthetic weighted mean patterns) in the EBA were relatively high. In total, this analysis was not very sensitive. Only in the EBA, could the whole person patterns successfully decode the synthetic mean patterns and synthetic weighted mean patterns. However, after FDR corrections for multiple comparisons, the result was no longer statistically significant. Only the results in the OFA, EBA, STS, and FG are shown in **Figure 7**, as accuracies in the other three ROIs were relatively small (see the detailed classification accuracies in **Supplementary Tables S1**, **S2**).

## Discussion

In the present study, we explored how specific ROI responses to the whole persons were associated with the responses to the isolated faces and bodies. Our MVPA and pattern similarity findings suggested that the whole person patterns approximated the combined weighted mean patterns of face and body in the EBA. Furthermore, the correlation coefficient of the body pattern was lower than that of the face pattern for happy expressions, although it was equal to that of the face patterns for the two threatening expressions (anger and fear).

### A Pattern Similarity between the Whole Person and Synthetic Person Was Shown in the EBA

In our study, the EBA and STS were the sole brain ROIs in which whole person–synthetic correlations were significantly greater than both whole person–face and whole person–body correlations irrespective of emotions. As a consequence, respective information from the face and body patterns contributed to the high similarity between the whole person patterns and the synthetic mean patterns in the EBA (Kaiser et al., 2014). The finding that the face and body patterns provided unique information to whole person patterns showed that the face and body were represented separately in the EBA. Developmental work has suggested that the response patterns of monkey inferior temporal neurons showed obvious clusters specific for faces, hands, and bodies (Kiani et al., 2007). Our findings were consistent with one functional imaging study that had proposed a distributed representation of faces, bodies, and objects in the human OTC, and highlighted category-specific modules in processing them at the same time (Caspari et al., 2014; Watson et al., 2016). Together with our findings, these results indicated that representations of faces and bodies in the EBA were likely to be quite distinct, supporting a part-based representation of whole persons therein.

Face and body patterns contribute equally in combination to form whole person patterns in the EBA for threatening expressions. In the EBA, the optimal weight of body patterns was lower than that of face patterns for happy expressions, but it was almost the same as that of face patterns for angry and fearful expressions. Faces and bodies are both familiar and salient in our daily life, and often convey some similar information, leading to many common points of processing even in affective neuroscience (de Gelder et al., 2010, 2015; Kim and McCarthy, 2016). Furthermore, unlike many studies using headless bodies, we employed bodies with blurred faces to avoid the confounder in which the headless bodies act as novel stimuli that attract more attention than normal. This ensured the contributions of faces and bodies combined to form the whole person would be compared fairly (Kret et al., 2011a,b). One previous study demonstrated that in the EBA, both faces and bodies produced more activations for threat than neutral expressions, and the difference in bodies versus faces was even larger (Kret et al., 2011b). Another study discovered that happy postures were less attended to than either angry or fearful postures by applying gaze measures (Kret et al., 2013). All the above findings were in accordance with our conclusions that body patterns might have a smaller weight than face patterns for happy expressions, but equal weights for angry and fearful expressions.

### Potential for Emotion Classification Performance by the Synthetic Weighted Mean Person in the EBA

In this study, we found that the responses to whole persons were potentially decoded by a weighted average of the responses to face and body, which was in line with previous studies mainly concerning object representations (Agam et al., 2010; Watson et al., 2016). Furthermore, other studies have found evidence for other forms of linear combinations (Zoccolan et al., 2005; Macevoy and Epstein, 2009) and for nonlinearities (Gawne and Martin, 2002; Heuer and Britten, 2002). Studies with monkeys can measure responses at the level of individual neurons, which is not practical in humans. A macaque study (Zoccolan et al., 2007) found that the exact relationship depended on how selective a neuron was for the given stimuli. For highly selective neurons, the relationship tended to be a simple average, as suggested in another experiment (Zoccolan et al., 2005) and which was in accordance with our study. Through a regression analysis, rather than MVPA, many studies of human subjects have found that a linear combination of the responses to two single objects could best decode the responses to the pair, supporting the most comprehensive model, the weighted mean of face and body patterns used in our experiment t (Macevoy and Epstein, 2009; Baeck et al., 2013).

Moreover, the body perception mechanism was directly explored in several studies (Droit-Volet and Gil, 2016; Borgomaneri et al., 2017). One previous study (Taylor et al., 2007) found a gradual activity increase in the EBA as more body information was represented, suggesting, as we found, that the whole person might be represented in a part-based manner in the EBA. Another study (Liu et al., 2010) found that the OFA was sensitive to face parts. Given the previously proposed functional analogy between face- and body-sensitive areas of the VOTC (Minnebusch and Daum, 2009), we speculated that EBA might represent whole persons in a part-based manner, whereas the OFA preferred face parts. In addition, a recent study (Brandman and Yovel, 2016) pointed out that whole bodies were presented in a configurable rather than part-based manner in two body-selective areas, the EBA and FBA, by comparing the whole bodies and sums of their scrambled parts. Some aspects of this discrepancy may be explained. The preceding study mainly focused on the first-order configuration, so the presentation of stimuli was different from ours. That is, all of the body parts were presented simultaneously in a scrambled manner in the foregoing study, while parts were presented as isolated faces and bodies in our study. What’s more, only signal changes and two-classification approaches were used in that study, resulting in a less comprehensive analysis to some extent. Furthermore, the emotion factor was not considered in any of the above studies. It is notable that some other studies, respectively, discovered that faces were represented in a holistic manner in the FFA (Zhang et al., 2012; Song et al., 2013), that configurable processing of headless bodies occurred in the right FBA (Soria Bauser and Suchan, 2015), and that a linear combination of face and headless body patterns was utilized in the FG (Kaiser et al., 2014). Our study showed no precise combination relationship in the FG, probably because not only face- and body-selective neurons, but also other neurons, were tuned uniquely to whole persons. This finding was confirmed in another study (Bernstein et al., 2014), which proposed that the integration of faces and bodies into whole persons was found in the FG at mid-level stages of object processing, but not in the lateral-occipital face and body areas at early stages. In our study, the face-selective areas (OFA and IFG) and emotion-sensitive areas (AMG and insula), as well as the STS, showed no part-based representation. The OFA has been reported to be capable of handling faces at the level of parts (Taylor et al., 2007; Liu et al., 2010). IFG, AMG, and insula could mainly process the information of emotional faces (Ishai et al., 2005; Fox et al., 2009). The results for the STS might originate from two sources. First, in our experiment, the STS may have lacked enough voxels sensitive to bodies; second, it may not have participated in the separate processing for faces and bodies, since it was reported to play a key role in integrating information from many channels (de Gelder et al., 2010; Candidi et al., 2015). To sum up, the current study is the first to apply pattern similarity analysis, weight analysis, and classification analysis to explore the linear relationship of emotion perception in faces, bodies, and whole persons in the AMG, IFG, OFA, EBA, STS, FG, and insula.

### Limitations

Several limitations should be addressed in this study. (1) Choice of the stimuli: in our study, we investigated whether there are brain regions that could be modulated by emotions when representing the whole person. However, as there is no neutral condition, our research is limited to a certain extent. Future work is needed to examine the differences within each brain region between positive emotional modulation and the modulation of relatively neutral emotions, in addition to the differences between negative emotional modulation and neutral emotional modulation, when neutral stimuli are included. (2) Sample of the study: to predetermine the sample size, *a priori* power analysis was conducted using the statistical software G ^∗^ Power^4^. Based on the literatures we referred, we first calculated the effect sizes in these studies which ranged from 0.29 to 0.96. We assumed that our study had a moderate effect size (ranged from 0.65 to 0.79). The required sample size was then computed with *a priori* power analysis, when α error probability was 0.05, power (1-β error probability) was 0.95, and the effect sizes changed from 0.65 to 0.79. The *a priori* power analysis suggested the required sample size was from 19 to 28 subjects. In our study, 20 subjects were included for further analyzes, which was not large enough. Although our sample size was similar to those reported in previous publications (Taylor et al., 2007; Prochnow et al., 2013; Kaiser et al., 2014; Brandman and Yovel, 2016) and one of our latest studies on the facial affective expression decoding (Liang et al., 2017), a larger group of participants was needed in the future studies. Moreover, when the sample size gets larger, a bigger statistical power can be obtained. And a larger number of participants can better prove the effectiveness of our findings, and separate truly significant results from apparent trends or false results related to having too few subjects. Furthermore, replicating this study with a larger number of participants, and examining the potential age-related differences between different age groups are also aspects of this issue worthy of study.

Several studies have shown that the body and face are processed separately in the early stages of processing (in the EBA and OFA, respectively), and then integrated into a representation in the FG. Therefore, each brain region may not be independent when perceiving the whole person, but instead may be somewhat dependent on each other. Our future work requires further exploration of the relationship between these brain regions associated with body perception and face perception, followed by construction of a larger brain area based on these relationships to reveal the underlying mechanisms when perceiving the whole person. Additional future work should identify whether there are brain regions representing whole person patterns in a more complex way, such as the second-order combination of faces and bodies. Furthermore, choosing weights for the synthetic weighted mean approach based on the similarity of the produced synthetic weighted mean patterns to the whole person patterns may introduce a bias in the classification. Future work is needed to develop the novel method of the weight analysis in calculating the synthetic weighted mean patterns to minimize the bias in the pattern classification analysis.

## Conclusion

This study provided tentative evidence that whole person patterns could be modeled by a linear combination of face and body patterns, and that there was emotional modulation in the EBA. Firstly, we found significant correlations between the whole person patterns and the synthetic mean patterns in the EBA for all three emotions. Secondly, the face and body patterns made equal contributions to integrating information when combining into whole person patterns for threatening expressions, while the face patterns shared a greater contribution for happy expressions. To summarize, we suggest that there are significant correlations in perceiving emotions expressed by dynamic faces, bodies, and whole persons. Furthermore, the human brain can perceive whole persons in a part-based manner in the EBA. Our study provided new evidence that emotions can modulate the correlations between different patterns. Future work is needed to examine the detailed functional interactions in representing emotions of whole persons in specific brain areas, and the differences between emotional modulation and the modulation of neutral conditions within each specific brain regions.

## Author Contributions

BL designed the experiments. XY, JX, and PW performed the experiments. XY, JX, and LC analyzed the results. XY and JX wrote the manuscript. JX, XL, and BW contributed to manuscript revision. All authors contributed to discuss the results and have approved the final manuscript.

## Conflict of Interest Statement

The authors declare that the research was conducted in the absence of any commercial or financial relationships that could be construed as a potential conflict of interest.

## Footnotes

1 http://www.fil.ion.ucl.ac.uk/spm/software/spm8/

2 http://www.csbmb.princeton.edu/mvpa

3 http://www.csie.ntu.edu.tw/~cjlin/libsvm/

4 http://www.gpower.hhu.de/

## Supplementary Material

**FIGURE S1 |** Results of multi-voxel pattern analysis in a standardized color scale. We also tried to standardize the color scale, but the difference between the patterns of the brain regions became insignificant.

**TABLE S1 |** Classification accuracies of the whole person predictor trained by the face, body, synthetic mean person and synthetic weighted mean person patterns. The chance level is 33.33%. AMG, amygdala; IFG, inferior frontal gyrus; OFA, occipital face area; EBA, extrastriate body area; STS, superior temporal sulcus; FG, fusiform gyrus; INS, insula.

**TABLE S2 |** FDR correction for multiple comparisons of the above table.
