# Supplementary figures and images for: Linear Representation of Emotions in Whole Persons by Combining Facial and Bodily Expressions in the Extrastriate Body Area
Source: Front Hum Neurosci. 2018 Jan 10;11:653. doi: 10.3389/fnhum.2017.00653 (PMC5767685; doi:10.3389/fnhum.2017.00653)

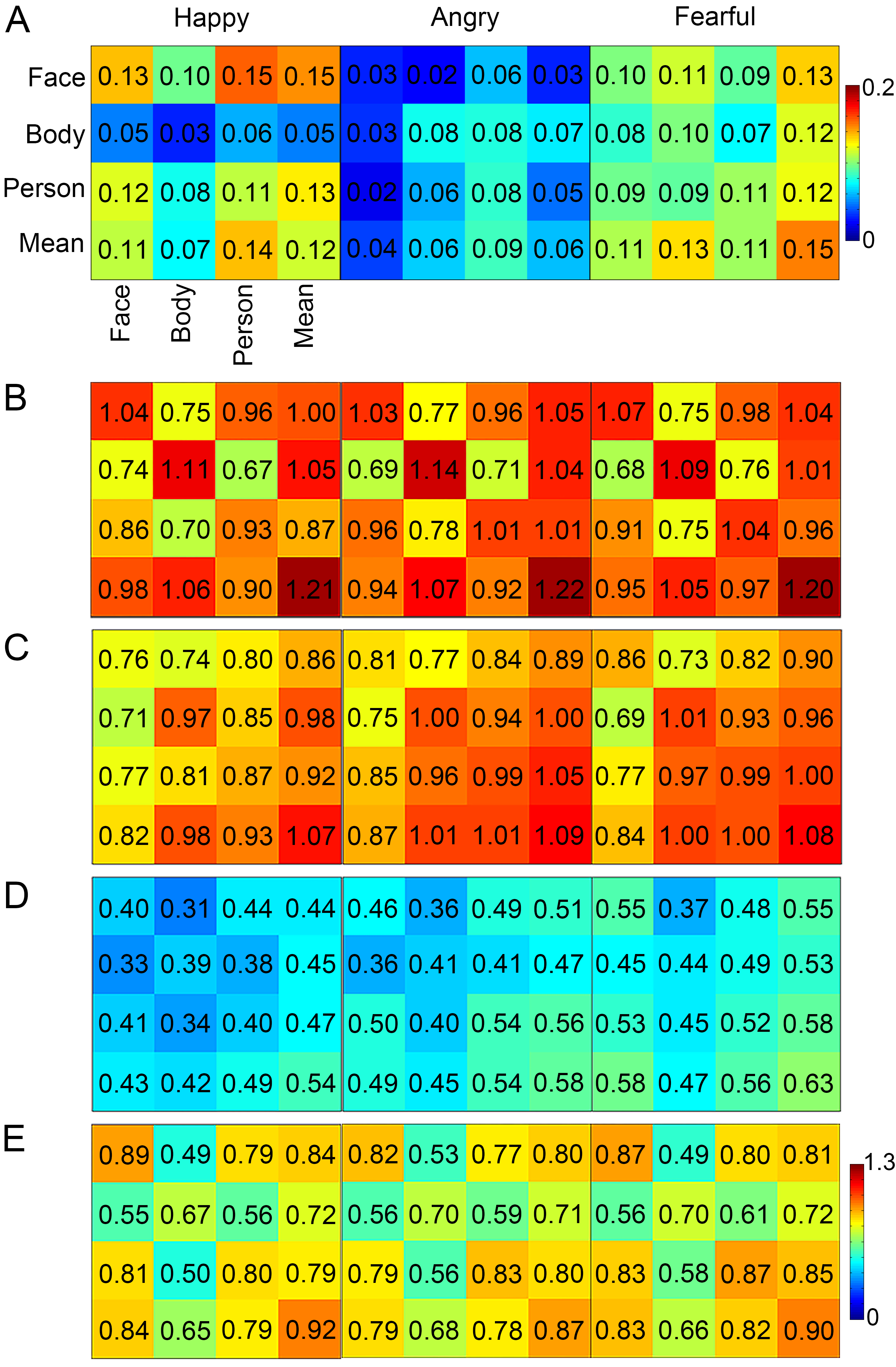

Supplement: Supplementary file 1 [file Image_1.TIF]
