# Supplementary material for: Linear Representation of Emotions in Whole Persons by Combining Facial and Bodily Expressions in the Extrastriate Body Area
Source: Front Hum Neurosci. 2018 Jan 10;11:653. doi: 10.3389/fnhum.2017.00653 (PMC5767685; doi:10.3389/fnhum.2017.00653)
Supplement: Supplementary file 2 [file Table_1.DOC]

**Table 1.** Classification accuracies of the whole person predictor trained by the face, body, synthetic mean person and synthetic weighted mean person patterns. The chance level is 33.33%.

| Brain areas | Classification accuracies (%) | | | | | | | |
| --- | --- | --- | --- | --- | --- | --- | --- | --- |
|  | Face | | Body | | Simple Mean | | Weighted Mean | |
|  | Mean | p | Mean | p | Mean | p | Mean | p |
| AMG | 32.85 | .390 | 33.07 | .772 | 32.82 | .539 | 32.67 | .403 |
| IFG | 32.97 | .521 | 33.04 | .732 | 33.83 | .478 | 34.31 | .140 |
| OFA | 33.19 | .826 | 31.01 | 0 | 32.47 | .245 | 34.53 | .188 |
| EBA | 34.85 | .089 | 33.24 | .906 | 35.15 | .032 | 35.74 | .020 |
| STS | 34.40 | .142 | 31.89 | .161 | 33.29 | .965 | 34.15 | .344 |
| FG | 33.21 | .876 | 31.92 | .033 | 33.42 | .904 | 34.08 | .307 |
| INS | 33.29 | .948 | 31.63 | .029 | 33.21 | .857 | 32.75 | .507 |

AMG, amygdala; IFG, inferior frontal gyrus; OFA, occipital face area; EBA, extrastriate body area; STS, superior temporal sulcus; FG, fusiform gyrus; INS, insula.

**Table 2.** FDR correction for multiple comparisons of the above table.

| Brain areas | Classification accuracies (%) | | | |
| --- | --- | --- | --- | --- |
|  | Face | Body | Simple | Weighted |
|  |  |  | Mean | Mean |
|  | p | p | p | p |
| AMG | .752 | .965 | .794 | .752 |
| IFG | .794 | .965 | .794 | .497 |
| OFA | .965 | 0 | .624 | .526 |
| EBA | .415 | .965 | .185 | .185 |
| STS | .497 | .501 | .965 | .741 |
| FG | .965 | .185 | .965 | .716 |
| INS | .965 | .185 | .965 | .794 |
